# Pharmacological Activities of Psoralidin: A Comprehensive Review of the Molecular Mechanisms of Action

**DOI:** 10.3389/fphar.2020.571459

**Published:** 2020-10-22

**Authors:** Javad Sharifi-Rad, Senem Kamiloglu, Balakyz Yeskaliyeva, Ahmet Beyatli, Mary Angelia Alfred, Bahare Salehi, Daniela Calina, Anca Oana Docea, Muhammad Imran, Nanjangud Venaktesh Anil Kumar, Maria Eugenia Romero-Román, Alfred Maroyi, Miquel Martorell

**Affiliations:** ^1^ Phytochemistry Research Center, Shahid Beheshti University of Medical Sciences, Tehran, Iran; ^2^ Science and Technology Application and Research Center (BITAUM), Bursa Uludag University, Bursa, Turkey; ^3^ Faculty of Chemistry and Chemical Technology, Al-Farabi Kazakh National University, Almaty, Kazakhstan; ^4^ Department of Medicinal and Aromatic Plants, University of Health Sciences, Istanbul, Turkey; ^5^ Department of Chemistry, Manipal Institute of Technology, Manipal Academy of Higher Education, Manipal, India; ^6^ Medical Ethics and Law Research Center, Shahid Beheshti University of Medical Sciences, Tehran, Iran; ^7^ Department of Clinical Pharmacy, University of Medicine and Pharmacy of Craiova, Craiova, Romania; ^8^ Department of Toxicology, University of Medicine and Pharmacy of Craiova, Craiova, Romania; ^9^ Faculty of Allied Health Sciences, University Institute of Diet and Nutritional Sciences, The University of Lahore, Lahore, Pakistan; ^10^ Laboratorio de Análisis Químico, Departamento de Producción Vegetal, Facultad de Agronomía, Universidad de Concepción, Concepción, Chile; ^11^ Department of Botany, University of Fort Hare, Alice, South Africa; ^12^ Department of Nutrition and Dietetics, Faculty of Pharmacy, Centre for Healthy Living, University of Concepción, Concepción, Chile; ^13^ Unidad de Desarrollo Tecnológico, UDT, Universidad de Concepción, Concepción, Chile

**Keywords:** *Psoralea corylifolia*, herbal medicine, psoralidin, bioavailability, biological properties, molecular mechanisms, cell signalling pathways

## Abstract

Analysis of the most relevant studies on the pharmacological properties and molecular mechanisms of psoralidin, a bioactive compound from the seeds of *Cullen corylifolium* (L.) Medik. confirmed its complex therapeutic potential. In the last years, the interest of the scientific community regarding psoralidin increased, especially after the discovery of its benefits in estrogen-related diseases and as a chemopreventive agent. Growing preclinical pieces of evidence indicate that psoralidin has anticancer, antiosteoporotic, anti-inflammatory, anti-vitiligo, antibacterial, antiviral, and antidepressant-like effects. Here, we provide a comprehensive and critical review of psoralidin on its bioavailability, pharmacological activities with focus on molecular mechanisms and cell signaling pathways. In this review, we conducted literature research on the PubMed database using the following keywords: “Psoralidin” or “therapeutic effects” or “biological activity” or “*Cullen corylifolium*” in order to identify relevant studies regarding PSO bioavailability and mechanisms of therapeutic effects in different diseases based on preclinical, experimental studies. In the light of psoralidin beneficial actions for human health, this paper gathers complete information on its pharmacotherapeutic effects and opens new natural therapeutic perspectives in chronic diseases.

## Introduction

Used since ancient times in traditional medicine, *Cullen corylifolium* (L.) Medik. (syn. *Psoralea corylifolia* L.) has proven its therapeutic properties over the centuries. One of the main bioactive compounds is psoralidin (PSO), a coumestan derivative with a lot of pharmacological properties (Chopra et al., 2013). A diversity of pharmacological activities of PSO compound has been identified and described. PSO exerts therapeutic effects on cardiovascular and inflammatory diseases (Chopra et al., 2013; Yan et al., 2015).

**FIGURE 1:**
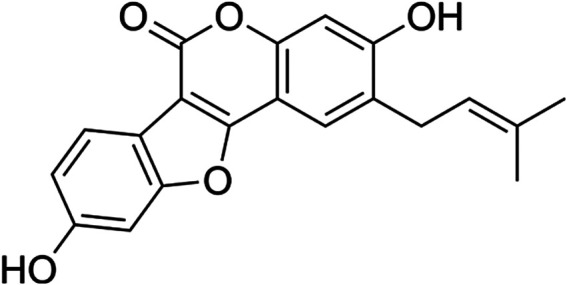
Chemical structure of psoralidin (3,9-dihydroxy-2-prenyl-coumestan).

PSO has a lot of beneficial health effects as an antibacterial against gram-negative and gram-positive germs (Borate et al., 2014), antioxidant (Xiao et al., 2010), anti-inflammatory modulating the inhibition of proinflammatory cytokines (Lee et al., 2012), antimycobacterial (Khushboo et al., 2010), antipsoriatic (Dwarampudi et al., 2012), and antidepressant (Yi et al., 2008). Besides, PSO possesses osteoblast proliferation-stimulating effects (Larsson and Fazzalari, 2014), estrogenic-like effects (Xin et al., 2019), and antitumor properties (Szliszka et al., 2011; Luo et al., 2013; Pal et al., 2017a; Badarau et al., 2019; Rusu et al., 2019). PSO is a natural phenolic coumarin in the same class with isopsoralen, psoralen, bakuchiol, bakuchalcone, flavones, and bavachinin (Zhang et al., 2016) isolated and identified from the seeds of the medicinal plant *Cullen corylifolium.* The plant is widely distributed in different regions of Asia, India and Europe (Zhang et al., 2019). PSO is a coumestan derivative with an isopentenyl group at the second carbon position of coumestrol (Liu et al., 2014) (Figure 1).

**FIGURE 2:**
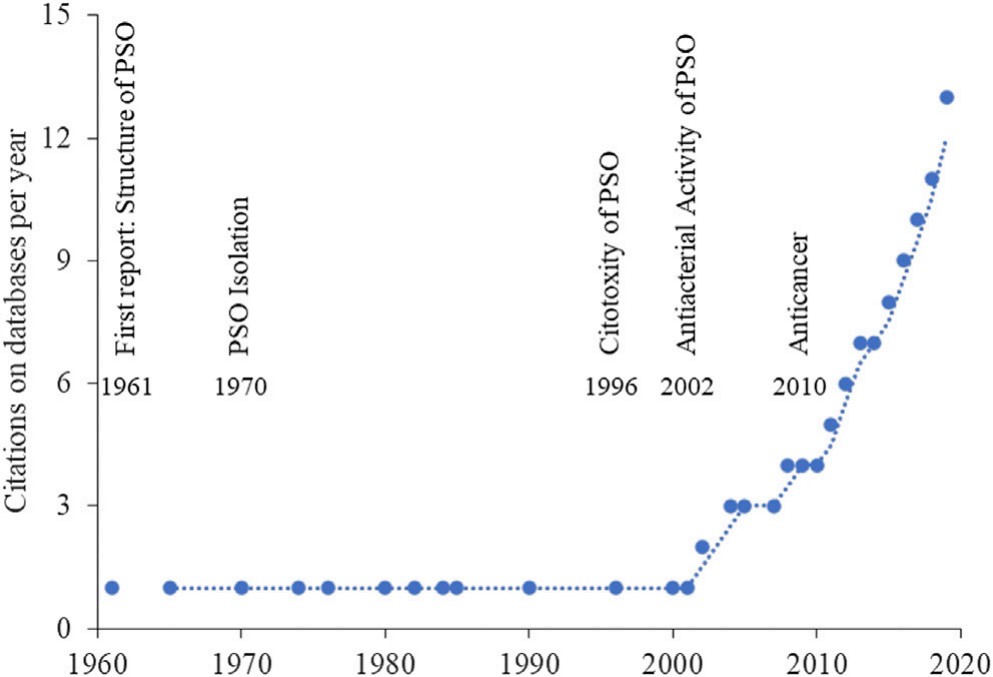
Psoralidin citations that appear along the time from 1961 till 2019. The Pubmed database was searched by the use of “psoralidin” as a keyword. The plot shows the cumulative number of hits identified for each year after the first report of psoralidin (PSO).

The insolubility of PSO in water is one of the main reason why *in vivo* studies are difficult to be conducted (Hao et al., 2014). However, dozens of studies have been performed (Figure 2) since 1961, when the first report of the structure of PSO was published by Khastgir (Xin et al., 2019). Initially, the studies focused on the evaluation of PSO cytotoxicity, but further *in vivo* showed its efficacy in preventing or reducing the progression of many pathologies.

The literature available in the PubMed database was researched for ethnomedicine uses, phytochemistry, pharmacological molecular mechanisms and PSO bioavailability by applying the following keywords: “*Psoralea corylifolium*,” “*Cullen corylifolium*,” “phytochemistry,” “bioavailability,” “anticancer,” “Anti-osteoporosis,” “Anti-inflammatory,” “antibacterial,” “SARS-CoV antiviral,” “neuro-protective,” “anti-vitiligo,” “antiprotozoal,” “vasodilatory,” “anti-apoptotic,” “antidepressant-like effects,” and “mechanism of action” their titles corresponding to the medical subject (MeSH) using OR/AND conjunctions. The research focused on studies in ethnomedicine, phytochemical, preclinical and clinical reports available to understand the mechanisms of action. Searches were limited to works *in extenso* in English and did not include studies with homeopathic preparations.

Very few clinical studies have been found in the literature on the efficacy of PSO, so more clinical research is needed in the future. Highlighting the molecular pharmacological mechanisms of PSO is the strength of this study because it can lead to the development of useful pharmaceutical formulations in the treatment of many chronic diseases.

## Bioavailability of Psoralidin

The bioavailability reflects the real available concentration at the site of drug action after absorption of these compounds from the gastrointestinal tract following oral administration of a dosage form (solution, suspension, tablet, capsule, powder, or elixir) (Chow, 2014; Salehi et al., 2019b). The percentage of a medicinal product absorbed unmodified in the systemic circulation represent the bioavailability of that product (Pandareesh et al., 2015; Salehi et al., 2020a).

PSO is an orally bioavailable natural compound. New pharmaceutical forms such as nanoencapsulation have been developed to increase the reduced bioavailability of PSO (Yi et al., 2008; Chen et al., 2017; Zhang et al., 2019). The results of Yin et al. (2016) demonstrate that nanoencapsulation of PSO (via chitosan and Eudragit S100) increased the oral delivery efficacy. By analyzing the pharmacokinetic profiles and some non-pharmacokinetic facings by the non-compartmental model of some suspensions of PSO and PSO-nanoencapsulated, it was found that the blood concentrations of the nanoencapsulated pharmaceutical forms are much higher at all moments of determination. The maximum concentration of nanoencapsulated PSO was 943 ng/ml compared to the simple PSO suspension whose concentration was 357 ng/ml, proving that nanoencapsulation favors the increased oral absorption of PSO more than conventional suspensions. Moreover, the peak time and the half-time remained the same, not being modified. The bioavailability of PSO in nanoencapsulated pharmaceutical forms was 339.02% higher compared to the control, a simple suspension with PSO. As a result, PSO pharmaceutical nanoformulations with chitosan and Eudragit S100 compounds have shown great potential for improving PSO bioavailability. It is still a need for further studies to improve the bioavailability of this promising compound.

## Pharmacological Importance of Psoralidin: *In Vitro* and *In Vivo* Studies

**FIGURE 3:**
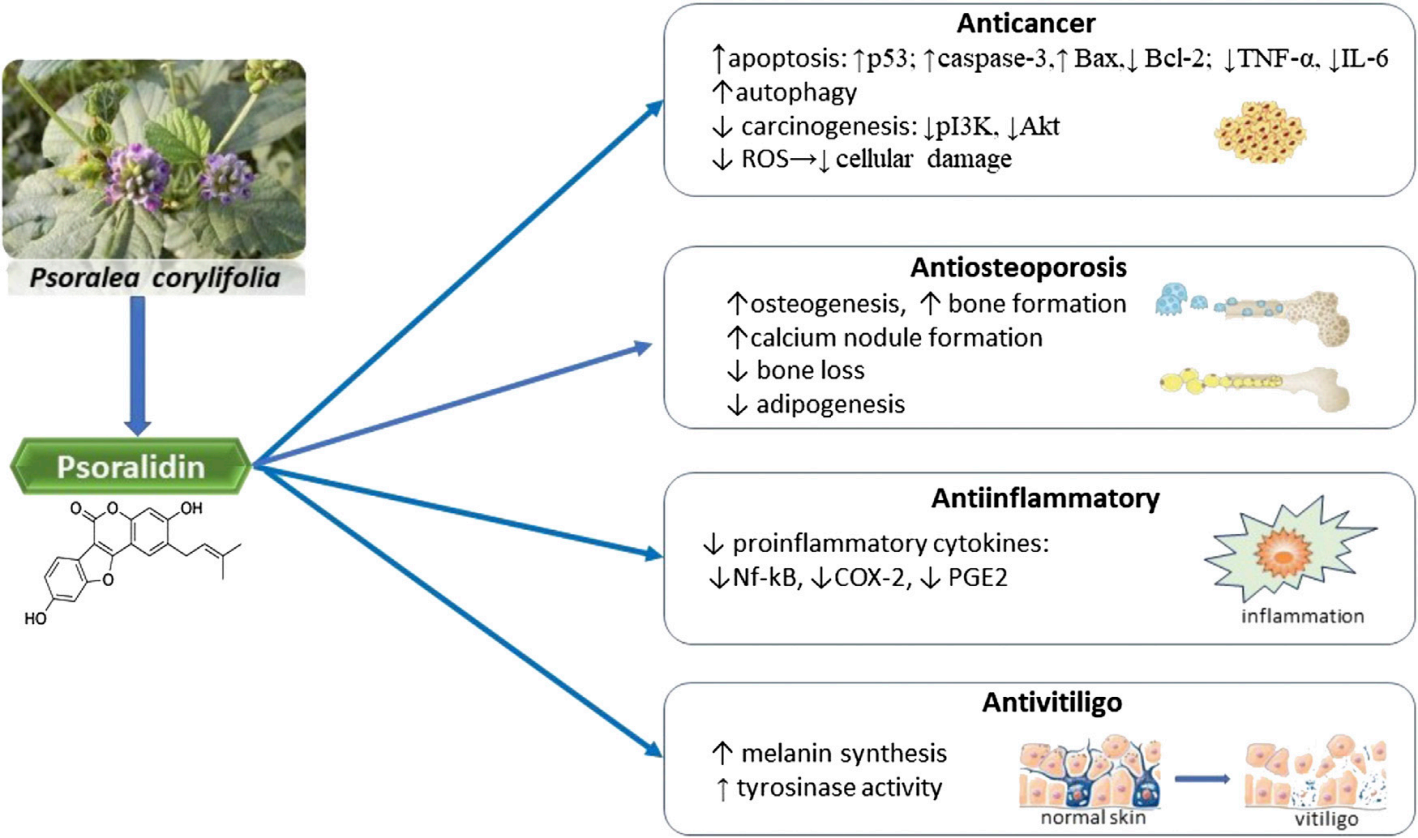
Summarized scheme of the main pharmacological activities of psoralidin. ↑, increase; ↓, decrease; NF-κB, nuclear factor kappa beta; COX, cyclooxygenase; PGE2, prostaglandin E2.

The studies have proven that PSO has the next pharmacological properties: antidepressant (Farahani et al., 2015), modulator of tumor necrosis factor-related apoptosis-inducing ligand (TRAIL) signaling pathway (Rana et al., 2014) and it is an potential estrogen receptor modulator (Yang et al., 2019). The main therapeutic properties of PSO and its mechanisms of action are summarized in Figure 3.

### *In Vitro* Studies

Cancer is one of the most feared diseases, and the fight against it has concentrated the efforts of thousands of medical researchers (Mishra et al., 2018; Salehi et al., 2019a). For hundreds of years, plants with anticancer effects have succeeded in improving the condition of the patients and, in some cases, to stimulate defense mechanisms of the body against certain types of cancer (Sharifi-Rad et al., 2020). It is not surprising that in recent years researchers have focused on the thesaurus of medicinal plant substances and have conducted research to identify compounds that are effective in anticancer therapies (Sani et al., 2017; Salehi et al., 2019a). PSO has been used in many *in vitro* models to evaluate its anticancerous effects and the underlying mechanisms (Xin et al., 2019). Determination of cytotoxicity after PSO contact with cells is done by calculating the percentage of dead cells and IC50 or the concentration of PSO that inhibited by 50% cell death (He et al., 2016).

PSO promotes cellular apoptosis and inhibits the cancer cells proliferation in prostate cancer (Szliszka et al., 2011; Das et al., 2014); breast cancer (Dwarampudi et al., 2012); liver cancer (Luo et al., 2013); colon cancer (Jin et al., 2016a); esophagal carcinoma (Jin et al., 2016b), or modulate the autophagy in the lung (Hao et al., 2014; Xin et al., 2019).

According to various studies, PSO has been recognized to inhibit forskolin-induced corticotrophin-releasing factor gene transcription (Chen et al., 2008). In a recent survey, it has shown potent *in vitro* activity against colon, gastric, breast and prostate cancer lines. It has been shown to exhibit a substantial effect on inhibiting protein tyrosine phosphatase 1B, a crucial metabolite involved in insulin signaling (Ren et al., 2019).

Different studies were conducted on the cytotoxicity of PSO suggested that it has shown the potential to induce cytotoxicity against breast (MCF-7) cancer, colon (HT-29), and gastric (SNU-1, SNU-16) cells. In prostate cancer cells, androgen-independent (DU-145, PC-3) androgen-dependent (LNCaP, C4-2B), the treatment with PSO also caused apoptosis. The growth of PC-3 xenograft tumor was also inhibited in nude mice by PSO (Srinivasan et al., 2010; Chiou et al., 2011; Hao et al., 2014).

Studies have been shown that PSO has a benefic role against diabetes complications, oxidative stress, obesity, osteoporosis, as well as on cell proliferation, autophagy, and apoptosis (Lim et al., 2011). PSO is implicated in the modulation of the autophagy process and inhibiting cadmium-transformed prostate epithelial cell xenograft growth thought placenta-specific 8 inhibition expression, reducing the levels nuclear factor kappa B (NF-κB) and B-cell lymphoma-2 (Bcl-2) expression and enhancing apoptotic gene expression (Pal et al., 2017a). Moreover, PSO has been found to decrease blood glucose, improve glucose tolerance and increase serum insulin levels (Seo et al., 2014).

PSO also has been found to promote the mineralization, suppress the adipocytes formation, increase the alkaline phosphatase activity, calcium nodule formation, and osteocalcin levels in bone marrow mesenchymal stem cells (BMSCs) whereas also down-regulated adipogenesis related factors. Moreover, PSO regulates the protein kinase B/glycogen synthase kinase 3β/β-catenin signaling pathway during the suppression of adipocyte differentiation (Cao et al., 2019). PSO significantly decreases cell proliferation and promotes apoptosis of Eca9706 cells in a dose-dependent manner. Moreover, it suppresses caspase-3 and NF-κB activities and also decreases the phosphatidylinositol-3-kinase (PI3K) and protein kinase B (Akt) protein expression dose-dependently (Jin et al., 2016b). Other *in vitro* studies on cell lines reported that PSO binds to both types of alpha and beta estrogen receptors, activating the classic estrogen receptor signaling pathway, thus explaining the effect of reducing bone loss as well as the antioxidant action (Liu et al., 2014). Furthermore, a very recent study has shown the dual beneficial antiosteoporotic effect of PSO by inhibiting adipogenesis and stimulating osteogenesis (Cao et al., 2019).

Researchers have used a mouse model of depression, and PSO has shown positive results in the forced swim test. 5-Hydroxyindole acetic acid and 5-hydroxytryptamine levels were significantly increased in the brain after PSO treatment, along with the changes in dopamine levels. Serum corticosterone, adrenal corticotropin-releasing hormone and corticotropin-releasing factor, hormones involved in stress control in mice, were reduced by PSO (Yi et al., 2008).

### *In Vivo* Studies

According to the animal studies carried out in till now, PSO has shown a variety of biological activities including antidepressant (Yi et al., 2008), anti-inflammatory (Yang et al., 2011), anti-osteoclastogenic (Kong et al., 2017), and anti-osteoporotic (Zhai et al., 2018) (Table 1).

**TABLE 1:** The most relevant information, including the preclinical pharmacological activities and mechanism of action of PSO.

Biologic effect	PSO tested	Model	Methods	Results/mechanisms	Reference
Anticancer	PSO/seeds	HeLa human cervical cancer cells/*in vitro*	Cytotoxicity: MTT assayCellular apoptosis: fluorescence microscopyDeath receptor expression TRAIL-R1, TRAIL-R2: flowcytometry	↑apoptosis, ↑TRAIL-R2 death, ↑mitochondrial damage synergistic effects: TRAIL 100 ng/ml + PSO 20/50 μMIC_50_ 20–50 μM	Bronikowska et al. (2012)
	PSO/(commercial sample)	SW480 human colon cancer cells/*in vitro*	Viability: MTT assayCellular apoptosis: DAPI staining assay, caspase-3 colourimetric assay, flow cytometric analysisNF-κB activity: ELISA, Western blot	↓NF-κB, p65, ↓Bcl-2, ↑Bax protein expression, ↑caspase-3↓cell viability, ↑apoptosisIC_50_ 20 μM	Jin et al. (2016a)
	PSO/(commercial sample)	A549, MCF-7 breast cancer cells/*in vitro*	Cancer cell proliferation: MTT assayDNA damage: comet assay autophagic vacuoles: MDCROS: a fluorescent probeNOX4: immunofluorescence staining	↑cell deaths, ↑autophagy↑DNA damage, ↓ROS generationIC_50_ 2.5–10 µM	Ren et al. (2016)
	PSO/(commercial sample)	Eca9706 esophageal carcinomaCells/*in vitro*	Cell proliferation, viability: MTTCellular apoptosis: flow cytometryApoptosis: DAPI staining assayCaspase-3 activity: colourimetric assayNF-κB: ELISA, Western blot	↓proliferation, ↑apoptosis↓PI3K, ↓AktIC_50_ 10–20 μM	Jin et al. (2016b)
	PSO/(commercial sample)	PC-3DU-145 prostate cancer cells/*in vitro*	Cell viability, apoptosis: MTT, luciferase assaysNF-κB: transcription factor assayIκB-α activation: ELISA, Western blot, kinase assays	↑phosphorylation of Akt in PC-3 (IC_50_ 60 μmol/L) and DU-145 (IC_50_ 45 μmol/L)↑PI3K in a dose and time-dependent manner. Selectively targets cancer cellsNo toxicity on normal prostate epithelial cells	Kumar et al. (2009)
	PSO/leaves	PC-3DU-145LNCaP4-2B prostate cancer cells/*in vitro*	Cell viability, apoptosis: MTT, kinase assay, Western blot analysis, ERK and JNK kinase assays, transient transfection and promoter assays immunohistochemical analysis using pEGFR (Tyr 1173) and pc-Jun antibodies	↓EGF-induced pEGFR expression in PC-3 and DU-145 cells↑EGF-mediated inhibition in PC-3 and DU-145↓JNK kinase activity, ↓prostate cancer growth. No toxicity↑EGFR activation, ↑apoptosis, ↓MAPK signaling, ↓cell proliferationIC_50_ 45–60 μM	Kumar et al. (2010)
	PSO/(commercial sample)	WPE-1 prostate epithelial cells/*in vitro*	Apoptosis: MTTProteins: Western blotNF-κB: RT-PCR	↓NF-kB signaling in cadmium-transformed prostate epithelial cellsIC_50_ 4 μM	Pal et al. (2017b)
	PSO/(commercial sample)	PC-3, DU-145 androgen-independent prostate cancer cell lines PzHPV-7 normal prostate epithelial cells/*in vitro*	Cell viability, apoptosis assays: MTTNF-kB, p65: ELISA, RT–PCR, Western blotCaspase-3 activation: fluorometryImmunohistochemistry: JC-1 staining	↓TNF-α↓NF-κB, ↑proapoptotic proteins↑caspase cascade→↑apoptosis↑death receptor-mediated apoptosisIC_50_ 45 μM	Srinivasan et al. (2010)
	PSO/(commercial samples)	NCA prostate cancer cells/*in vitro*	Cytotoxicity: MTT, LDH assaysApoptosis: fluorescence microscopy	↑apoptosis, ↓COX-2, ↓NF-kBIC_50_ 100 μM	Yang et al. (2011)
	PSO/seeds methanol extract	Mouse Hepa 1c1c7 cells/*in vitro*	Cytotoxicity: measuring cell survival using the crystal violet staining	Chemopreventive effectIC_50_ 0.5 μg/ml	Lee et al. (2009)
	PSO/seeds extract	MCF-7 human breast cancer cell linesIshikawa endometrial cancer cell line/*in vitro*	Estrogenic activity: molecular docking	↑endogenous estrogen-responsive gene ↑pS2↑ER-signalling pathwayClinical importance: novel estrogenic modulator. IC_50_ 10 μM	Liu et al. (2014)
	PSO (commercial samples)	ALDH, ALDHþ breast cancer stem cells/*in vitro*	Cell viability, apoptosis, colony formation, invasion, migration, small interfering RNA transfectionAldefluor assay for separation of the ALDH population: flow cytometry	↓growth of cancer cells↑apoptosis, ↓NOTCH1 signaling↓β-catenin and vimentin↑E-cadherin →↓migration, ↓invasionIC_50_ 0.5 μM	Sun et al. (2016)
	PSO/seed methanol extract	HT-29 colon MCF-7 breast human cancer cellsA541 (lung) hepG2 (liver hepatoma) cancer cells/*in vitro*	Cytotoxicity: MTT assay	↑cytotoxicity against HT-29 (colon) and MCF-7 (breast)PSO was not active against the A541 (lung) and HepG2 (liver hepatoma) cellsIC_50_ 0.3–0.4 μg/ml	Mar et al. (2001)
	PSO/(commercial sample)	Human liver microsomes, human intestine microsomesExpressed UGT enzymes/*in vitro*	Kinetic evaluation: Michaelis–Menten modelQuantification of glucuronides: UPLC analyses, immunoblotting protein levels: Western blot	Strong correlation PSO-3-*O*-glucuronidation and UGT1A9PSO-3-O-glucuronidation: *in vitro* marker for UGT1A9IC_50_ 5 μM	Sun et al. (2015)
	PSO/seeds	HeLa cells/*in vitro*	Cytotoxicity: MTT, LDH assays	Apoptosis, ↑expression of TRAIL-R2, death receptor, mitochondrial membrane potential, ↑cytotoxicity, ↑apoptosisIC_50_ 20–50 μM	Bronikowska et al. (2012)
	PSO/seeds	hepG2 human liver cancer cells/*in vitro*	Viability: MTTApoptosis: flow cytometryProteins: Western blot	↓viability, ↑activities of caspase-3, -8, and -9, ↑p53↓pro-survival genes Bcl-2, Bcl-xL↓caspase-3 proteinIC_50_ 64 μM	Yu et al. (2019)
Anti-osteoporosis	PSO/whole plantPSO/(commercial sample)PSO/seeds	Bone marrow mesenchymal stem cells (bmscs)Preosteoblast MC3T3-E1 cells preadipocyte 3T3-L1 cells/*in vitro*Newborn Sprague-Dawley rats/*in vivo* rat calvarial osteoblasts/*in vitro*Ovariectomized rats/*in vivo*	Cytotoxicity: MTT, LDH assaysProteins: Western blotAlkaline phosphatase: colourimetric assayOsteogenesis-related genes: qPCROsteogenic proteins: Western blot, ELISATRAP activity: Colourimetric assayDEXA, biomechanical analysis, tomographyProteins: Western blot	MC3T3-E1 cells: ↑osteogenesis, ↓adipogenesis, ↑calcium nodule formation, ↑alkaline phosphatase, ↑osteocalcin3T3-L1 cells: ↓adipocyte formation, ↓mRNA, ↓protein synthesis, ↑osteogenesis via mediating classical ER pathwayIC_50_ 1–10 μM↑osteoblasts proliferation, ↑differentiation↑ROB cell proliferation, ↑ALP activities, ↑calcified nodules, ↓COX-2, ↓ROS, ↑bone formation, ↑osteoblasts, ↓bone resorption of osteoclastsIC_50_ 10^–6^ mol/L↑bone formation↓bone resorption	Cao et al. (2019) Zhai et al. (2017) Zhai et al. (2018)
	PSO/(commercial sample)	BBMMs bone marrow macrophages/*in vitro*	Viability, apoptosis: MTT, LDH and DAPI stainingBone proteins: Western blot	↓TRAP-positive osteoclasts↓p-ERK, ↓p-38, ↓p-JNK↓NF-κb, ↓c-Fos/NFATC1, ↓TRAP, ↓cathepsin K, ↑RANKL, ↑OPGIC_50_ 0.1–30 μM	Kong et al. (2017)
Anti-inflammatory	PSO/fruits	Murine macrophages/*in vitro*	Neutrophil proinflammatory response: monitoring the inhibition of superoxide anion generation and elastase release superoxide anion generation: SOD-inhibition reductionNitrite concentration: ELISAViability: MTT	↓NO generation by murine macrophages in response to LPSIC_50_ 27, 46 μM	Chen et al. (2017)
	PSO/seeds	LPS-activated RAW264.7 cells/*in vitro*	NO synthesis: RT-PCR, Western blot	↓PI3K/Akt, ↓LPS induced iNOS expressionIC_50_ 1–30 μM	Chiou et al. (2011)
	PSO/(commercial sample)	Human normal lung fibroblasts/*in vitro*	Viability: MTT, Western blot, luciferase reporter gene assay, cell migration assay	↓IR-induced COX-2, ↓PGE2 ↓PI3K/Akt, ↓NF-κB↓proinflammatory cytokines (TNF-α, TGF-β, IL-6, IL-1 a/b)IC_50_ 50–100 μM	Yang et al. (2011)
Antibacterial	PSO/seeds	*S. aureus, S. epidermidis, P. aeruginosa, K. pneumoniae, P. mirabilis, P. vulgaris, E. coli*/*in vitro*	Diffusion method	↓bacterial grow at 1× 10^6^ cfu/mL stronger activity against Gram (−)Maximum inhibition against *K. pneumonie*	Borate et al. (2014)
Antiviral against SARS-CoV	PSO/dried seeds ethanol extract	SARS-CoV/in silico	Kinetics of enzymes: Lineweaver–Burk plots	Reversible mixed type I mechanisms	Kim et al. (2014)
Neuro-protective	PSO/seeds ethanol extract	BV-2 microglial cellsHT22 mouse hippocampal cells/*in vitro*	NO assay	Anti-neuroinflammatory↓LPS-induced NO production in BV-2 cells	Kim et al. (2016)
Anti-vitiligo	PSO/ethanol extract	Tyrosinase/*in silico*	HPLC tyrosinase activity: oxidation rate of levodopa assay	↑tyrosinase, a rate-limiting enzyme of melanogenesis	Shi et al. (2018)
Antiprotozoal	PSO/methanol extract	*Ichthyophthirius multifiliis*/*in vitro*	Bioassay-guide isolation and identification of active compounds efficacy assay of fractions against *I. multifiliis*	Detrimental effect on I. Multifiliis trophont *in situ*	Song et al. (2015)
Vasodilatory action	PSO/seeds ethanol extract	Male Sprague-Dawley rats/*in vivo*	Isometric tension recordings of rat aortic ringsIonic currents through TRPC3PSO concentration extract: 10–600 μg/ml	↑vasodilatation↑NO/cGMP↑prostaglandins	Gebremeskel et al. (2017)
Anti-apoptotic	PSO/seeds	Sprague-Dawley rats/*in vivo*	Annexin V/propidium iodide double-labelling fluorescence-activated cell sorting analysis	Protected rat chondrocytes from IL-1β-induced apoptosis, ↑Bcl-2↓Bax, ↓caspase-3, ↓caspase-9, ↓MMP-1, ↓MMP-13, ↑ ROS, ↑NO, ↑NF-κb	Rao et al. (2018)
Antidepressant-like effects	PSO/seeds	Male mice/*in vivo*	Open-field test in miceACTH, corticosterone: enzyme immunoassay	↓immobility time↑swimming behavior↑5-HT, ↑5-HIAA, ↑DA↓CRF, ↓ACTH, ↓corticosterone	Xu et al. (2008)

↓, Decrease; ↑, increase; 5-HIAA, 5-hydroxyindoleacetic acid; 5-HT, 5-hydroxytryptamine; ACTH, adrenocorticotropic hormone; Bcl-xL, B-cell lymphoma-extralarge; BMMs, bone marrow macrophages; BMSCs, bone marrow mesenchymal stem cells; COX-2, cyclooxygenase 2; CRF, corticotropin-releasing factor; CYP450, cytochrome P450; DA, dopamine; DAPI, 4′,6-diamidino-2-phenylindole; EGFR, epidermal growth factor receptor; ERK, extracellular-signal-regulated kinase; IκB-α, nuclear factor of kappa light polypeptide gene enhancer in B-cells inhibitor alpha; IL, interleukin; JNK, jun N-terminal kinase; MAPK, mitogen-activated protein kinase; MDC, autofluorescent compound monodansylcadaverine; MTT, 3-(4,5-dimethylthiazol-2-yl)-2,5-diphenyltetrazolium bromide; NF-κB, nuclear factor kappa B; NFATC1, nuclear factor of activated T cell cytoplasmic 1; NO, nitric oxide; NOTCH1, notch homolog 1 translocation-associated; NOX4, NADPH oxidase 4; LC-MS/MS, liquid chromatography–mass spectrometry; LDH, lactate dehydrogenase; LPS, lipopolysaccharide; OPG, osteoprotegerin; PI3K/Akt, phosphatidylinositol 3 kinase; PSO, psoralidin; RANKL, receptor activator for NF-κB; ROB, rat calvarial osteoblasts; ROS, reactive oxygen species; RT-PCR reverse transcription polymerase chain reaction; SAPK, stress-activated protein kinases; SARS-CoV, severe acute respiratory syndrome coronavirus; SOD, superoxide dismutase; TGF-β, transforming growth factor-beta; TNF, tumor necrosis factor; TRAIL, tumor necrosis factor-related apoptosis-inducing ligand; TRAP, tartrate-resistant acid phosphatase; TRPC3, transient receptor potential cation channel subfamily c member 3; UGT, UDP-glucuronosyltransferase; UPLC, ultra-performance liquid chromatography.

Yi et al. (2008) investigated the effect of PSO against depression in male mice. In this study, the forced swimming test was used to screen the antidepressant effect of PSO. PSO in doses 20, 40, and 60 mg/kg was administered to mice using as vehicle water 15 ml/kg by gastric gavage. The results revealed that administration of PSO significantly decreased immobility time and increased swimming behavior without altering climbing behavior in the mouse forced swimming test. Locomotor activity studied by the open-field test was not affected by PSO.

Moreover, the levels of monoamine neurotransmitters involved in the pathophysiology of depression as 5-hydroxytryptamine and 5-hydroxyindoleacetic acid (5-HIAA, the main metabolite of serotonin) increased significantly in various brain regions after PSO treatment. In mice exposed to forced swimming test, striatum dopamine levels were also changed. In mice, PSO also suppressed the increased level of serum corticotropin-releasing factor, adrenal corticotropin-releasing hormone and corticosterone concentrations induced by swimming stress. Overall, the results obtained in this study suggested that PSO antidepressant properties are mediated via the hypothalamic-pituitary-adrenal axis and monoamine neurotransmitter systems (Yi et al., 2008).

Inflammation acts as a significant limiting factor in the radiotherapy (Buga et al., 2019). In a study carried out a few years ago (Yang et al., 2011), the PSO (5 mg/kg) anti-inflammatory effect was investigated in IR-irradiated mouse lung (20 Gy at a dose rate of 0.81 Gy/min, for 12 h or 1 week). Twelve hours after treatment, PSO suppressed the radiation-increased mRNA levels of TNF-α, TGF-β, and ICAM-1 by half, and inhibited IL-6 and IL-1 α/β by one quarter. The PSO inhibitory effects were found to be more effective 1 week after irradiation. In conclusion, data obtained in this study suggested that PSO may regulate the inflammation process in the ionizing radiation-irradiated lung (Yang et al., 2011).

Regarding the osteoporosis, it occurs primarily in women with premature menopause, due to estrogen hormone deficiency (Salehi et al., 2020b). In the absence of this hormone, disorders occur in the assimilation and fixation of calcium which leads to weakening of the bones over time (Larsson and Fazzalari, 2014). Hormone replacement therapy has minimal indications due to severe side effects (thromboembolism, stroke, endometrial cancer, breast cancer, and cardiovascular disease) (Tsatsakis et al., 2019). Therefore, research has focused on finding natural estrogen analogues with more excellent therapeutic safety (He et al., 2017).

The similar structure of PSO with coumestrol, a phytoestrogen, allows PSO to bind efficiently to estrogen receptors. *In vivo* studies performed in ovariectomized rats showed that PSO could inhibit bone resorption of osteoclasts and stimulate the bone formation (Zhai et al., 2017).

In another study performed by Kong et al. (2017), the impact of PSO on osteoclastogenesis and bone loss was investigated in mice. A low (5 mg/kg) and high dose (50 mg/kg) PSO was administered orally before LPS injection (5 mg/kg) and then every other seven days. The femurs of mice were collected on day 10. The results showed that PSO treatment inhibited LPS-induced bone loss and osteoclastogenesis formation and markedly down-regulated inflammatory cytokines (TNF-α, IL-1β, and IL-6) expression.

In a similar study performed by Zhai et al. (2018), osteotropic activities of PSO from *Cullen corylifolium* and coumestrol from *Medicago sativa* L. were studied. PSO and coumestrol (at doses of 10 mg/kg body weight/day each) were administered to ovariectomized rats, and after 12 weeks of intervention, samples were collected. Both PSO and coumestrol suppressed ovariectomized bone loss *in vivo*. It was demonstrated that PSO suppresses bone loss and have better osteoprotective effects than coumestrol.

## Clinical Studies

Regarding human clinical trials with PSO, there is a scarcity. There are only a few that have included a reduced number of patients with dermatological conditions such as acne and vitiligo.

A small clinical study included 76 adolescent and young patients diagnosed with grade II and III acne vulgaris, who received double medication for 1 month: oral tablets that had as active principle PSO from *Cullen corylifolium* and topical cream Clarine with antimicrobial, anti-inflammatory, healing, antioxidant, astringent, and emollient properties. The results showed that patients with grade II acne had an excellent response (56.3% of cases) and good response (43.8% of cases). Patients with grade III acne had a good response (only 38.3% of cases) and a good response in (56.7% of cases). Thus, the combination of the oral and topical preparations had a synergistic, beneficial anti-acne effect (Gopal et al., 2001; Khushboo et al., 2009).

In another study, the combination of PSO with trioxalene (a chemical derivative) and the exposure to solar ultraviolet is an effective psoriasis treatment. Another clinical study included 30 patients with lip vitiligo who topically applied an ointment with *Cullen corylifolium* as the main ingredient. Patients with mild forms of vitiligo showed maximum improvement in 1–10 months, while chronic cases with lip vitiligo showed an inadequate response. Exposure of the patient to sunlight (5–30 min daily) with oral administration of this for 1–7 weeks gave improved the beneficial effects (Sharma et al., 2005). Possible mechanism of action: PSO interacts with ultraviolet in the epidermis forming photosensitive fragments of DNA. The maximum activity is at a wavelength of 325 nm having as effects: 1) slowing down the proliferation of keratinocytes; 2) suppression of the immune skin reaction; and 3) affects melanocytes, fibroblasts, and endothelial cells (Chopra et al., 2013).

Although there are many treatments for vitiligo, none work unanimously for all patients. Patients with vitiligo have areas with completely pigment-free skin. PUVA phototherapy (PSO + UVA) has proven to be effective over time, but narrow-band UVB therapy is gaining ground as a safe and effective therapeutic option. A recent study aimed to statistically compare the effectiveness of PUVA and nb-UVB treatments (type B ultraviolet, narrow-band). A group of 69 vitiligo patients who were treated with PUVA or nb-UVB were retrospectively analyzed. The two groups of patients were compared regarding the stage of repigmentation, the number of treatments until complete repigmentation, the appearance of newly affected areas or the increase in the size of existing ones, adverse effects of therapy, stability of repigmentation, and matching of pigment color. It has been shown that in the group treated with PUVA (31 patients), nine showed complete repigmentation (23.6%) and 14 a moderate improvement (36.8%), while in the group of those treated with nb-UVB (31 patients), 13 showed complete repigmentation (41.9%), and a moderate improvement (32.2%). Increased stability of repigmentation and better matching of pigment color to the nb-UVB-treated group was observed. The conclusions of the study were that nb-UVB is a more effective therapeutic method and the repigmentation induced by nb-UVB is statistically more stable (Parsad et al., 2006).

## Discussion

The strong point of this study was that many systematic reviews were included that highlighted the mechanisms of PSO pharmacological activities in many chronic diseases. Thus, the article provides an up-to-date overview of the potential therapeutic applications of this natural compound.

Although, this study presented the beneficial pharmacological activities of PSO for human health, but the main clinical therapeutic limitation derives from low absorption. Several methods have been tried to improve PSO absorption, of which prenylation has been shown to be the most effective, as prenylated metabolite it has to be more bioavailable, increasing estrogenic activity and thus improving the anti-osteoporotic effects (Kretzschmar et al., 2010; Mukai et al., 2013) and antioxidant (Li et al., 2014).

Psoralidin is a prenylated coumestans derivative, with multiple therapeutic effects proven in preclinical studies, of which the most important and with the greatest clinical applicability as adjuvant therapies are: antiosteoporotic and anticancer.

Osteoporosis is a condition that manifests itself by decreased bone mass and damage to bone tissue. Thus, even bone fractures can occur as a result of minor traumas, the most affected being the spine, hip, forearm, ribs and hand joint (Salehi et al., 2020b). PSO has osteoprotective effects, promotes osteogenic differentiation, mineralization of osteoblasts, inhibits bone resorption by reducing osteoclast differentiation.

Cancer is a malignant tumor (Zlatian et al., 2015). Tumors appear when cells start to proliferate uncontrollably and can be benign or malignant (Elsebai et al., 2016). Unlike benign tumors, malignant tumors can differentiate (and lose their normal function), having the ability to invade neighboring tissues and to metastasize, that means to form tumors secondary to other parts of the body than the site of origin (Buga et al., 2019).

Carcinogenesis is a multi-stage and multi-factorial process during which profound changes occur, allowing cells to proliferate uncontrollably and ultimately invading other tissues. This process can be described in three major stages: initiation, promotion, and tumor progression. The results of our study showed that PSO could reduce the proliferation of malignant cells and stimulate apoptosis, thus being a potential adjunct in anticancer treatment.

Viral infections play an important role in human disease, and the recent COVID-19 pandemic in the emergence of globalization and ease of travel have highlighted their prevention as a critical issue in protecting public health (Alam Riaz et al., 2020; Docea et al., 2020; Goumenou et al., 2020; Tsatsakis et al., 2020). Despite advances in immunization and drug development, many viruses lack preventive vaccines and effective antiviral therapies, which are often caused by the generation of viral escape mutants (Calina et al., 2020a; Calina et al., 2020b). Thus, the identification of new antiviral alternative treatments is of critical importance, and bioactive natural compounds are an excellent source for such discoveries (Sarkar et al., 2020). In a recent study, it was shown that ethanolic extract from *P. corylifolia* seeds has good activity against papain-like protease (PLpro), an essential enzyme involved in SARS-CoV replication. The bioassay-guided fractionation of this extract identified six polyphenolic compounds as the bioactive compounds responsible for action against SARS-CoV PLpro. Of these, the most potent compounds that inhibited SARS-CoV PLpro were PSO and isobavachalcone (with IC50 ranging from 4.2 to 38.4 μM). The anti-SARS-CoV-2 mechanism is a type I, whereby PSO binds selectively to the free enzyme and not to the enzyme substrate. Based on these preliminary results it can be considered PSO as being one of the natural compound as potential agents against coronaviruses (Kim et al., 2014; Mani et al., 2020).

Although promising preclinical pharmacological studies have highlighted the mechanisms underlying PSO use as adjunctive therapy, long-term toxicity studies and data on possible interactions with other drugs are required. Besides, additional clinical trials are needed to confirm the clinical utility in medical practice.

## Conclusion

The numerous bioactivities of PSO are summarized in this review outlining research approaches focusing on pharmacological activities and clinical trials. PSO has been proved to be a therapeutic option for cancer treatment. Based on this extensive research, we also suggest that PSO has potential as an antidepressant, anti-inflammatory, anti-osteoclastogenic, anti-osteoporotic and also bring beneficial effects in estrogen-related diseases. Regarding the bioavailability, we describe PSO as a non-toxic but low oral bioavailability compound; thus, new and further researches regarding the bioavailability of PSO as well as studies on pharmacokinetic are still required to improve the efficacy and delivery of PSO. The various therapeutic effects of PSO support its use as a potential drug in a wide range of medical conditions. Further research and studies are needed to confirm its benefits and efficacy in pharmaceutical formulations.

## Author Contributions

JS-R, MM, and DC: conceptualization. SK, BY, AB, BS, and MAA: validation investigation. DC, AD, NA, and MM: resources. MI, NA, MERR, and JS-R: data curation. MM, DC, BS, AM, and JS-R: review and editing. All authors: writing. All authors read and approved the final manuscript and contributed equally to the manuscript.

## Conflict of Interest

The authors declare that the research was conducted in the absence of any commercial or financial relationships that could be construed as a potential conflict of interest.
